# The failure pattern for the magnetic sphincter augmentation device: a single-institution case series with literature review

**DOI:** 10.1007/s00464-025-11842-x

**Published:** 2025-06-26

**Authors:** Samuel J. Bloomsburg, Anthony J. Duncan, Sugong Chen

**Affiliations:** 1 School of Medicine & Health Sciences, University of North Dakota, 1301 N Columbia Rd Stop 9037, Grand Forks, ND 58202-9037 USA; 2 Department of Trauma and Acute Care Surgery, Sanford Medical Center Fargo, 5225 23rd Ave. S., Fargo, ND 58104 USA; 3 Department of Bariatric Surgery, Sanford Medical Center Fargo, 5225 23rd Ave. S., Fargo, ND 58104 USA

**Keywords:** LINX removal, LINX failure pattern, LINX slippage, Magnetic sphincter augmentation, Hiatal hernia, GERD

## Abstract

**Background:**

Magnetic sphincter augmentation (MSA) is an effective surgical treatment for GERD. Removal rates are reported around 4–6%, with dysphagia being the most common indication for removal. This rate has increased over time, and the pattern of device failure has not been well established. This study characterizes a consistent pattern of MSA slippage or migration and contextualizes this within the existing literature on MSA failure.

**Methods:**

This is a single-institution retrospective review of patients who underwent MSA placement and device removal between 2014 and 2024. Radiographic and endoscopic images and operative reports were reviewed for the presence of pre-operative and post-operative hiatal hernia or device malposition. We also conducted a comprehensive review of existing literature on MSA explantation, with particular attention to device slippage, malposition, and hiatal hernia.

**Results:**

42 patients underwent MSA placement at our tertiary academic institution. Twelve of these, plus one patient who had MSA placed elsewhere, underwent device removal for symptoms of dysphagia and/or recurrent reflux (28%) with a median follow-up of 41.1 months. Ten of these 13 patients showed evidence of MSA slippage and/or hiatal hernia. In comparison, our literature review revealed explant rates of 0–12.6% (median 4.7%) with overall shorter terms of follow-up. Similarly, the most common reason for explantation was dysphagia, followed by recurrent or persistent reflux. However, device migration/hiatal herniation was a rare finding.

**Conclusion:**

In the long-term follow-up of MSA patients with dysphagia or recurrent reflux, we observe a pattern of device slippage or migration. This pattern mirrors the failure pattern of the 360-degree fundoplication. We suspect an under-appreciation of device slippage or migration as the etiology for these symptoms. While MSA is effective, continued improvements on implantation technique, coupled with careful patient selection and lifestyle counseling, may increase its long-term success rate.

**Graphical abstract:**

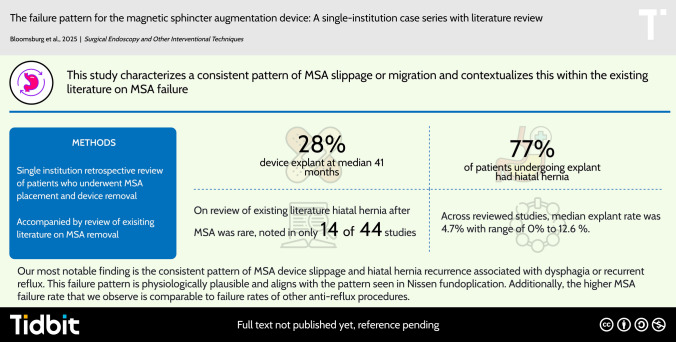

**Supplementary Information:**

The online version contains supplementary material available at 10.1007/s00464-025-11842-x.

Gastroesophageal reflux disease (GERD) is a common condition and is associated with considerable disease burden. First-line treatment consists of diet and lifestyle modification along with anti-secretory medication. Many patients ultimately require surgical intervention due to poor control, treatment intolerance or medication side-effects. Fundoplication combined with a good hiatus repair remains the gold standard for the surgical treatment of GERD. Magnetic sphincter augmentation (MSA) is a recently developed intervention for GERD [1]. This has enjoyed considerable success, demonstrating control of GERD comparable to laparoscopic fundoplication with a shorter and less complex procedure, quicker recovery, and less risk for the unfavorable sequelae of fundoplication such as gas bloat or inability to burp [2, 3]. The LINX Reflux Management System (Ethicon, Johnson and Johnson; Shoreview, MN) used for MSA consists of a ring of magnetic beads which is surgically implanted around the lower esophageal sphincter (LES) in order to augment LES tone [1]. More than 40,000 LINX devices have been implanted since 2012, and the increasingly long-term data continue to show device safety and effectiveness [4]. Nonetheless, a large portion of the foregut surgery community continues to be skeptical of the fate of the device, drawing from negative experiences from historical non-relaxing sphincter augmentation devices such as the Angelchik device. Therefore, ongoing outcome assessment is essential for enhancing the understanding of the device characteristics and improvement of the best practice guidelines, which may help support its broader use in appropriate patient populations. In a series of patients presenting for device removal due to dysphagia or recurrent GERD symptoms at our institution at long-term follow-up, we observed a consistent pattern of device slippage or migration, often with recurrent or de novo hiatal hernia. Furthermore, we conducted a literature review regarding MSA complications and explantations to help elucidate the pattern of MSA failure.

## Methods

### Case series

This was a retrospective review of patients at our academic, tertiary-care facility from the years 2014 to 2018 for implantation or 2024 for explanation. We identified all patients who underwent Magnetic sphincter augmentation (MSA) implantation and subsequently underwent MSA explanation. The study underwent IRB review (IRB: STUDY00003160, February 9, 2023). Data were collected from manual chart review of the electronic medical system. Data points collected included age at time of implant, sex, BMI, pre-operative DeMeester score and manometric data, hiatal hernia repair at time of implant, indication for removal, time until explant, and hiatal hernia at time of explant.

### Literature review

We conducted a literature review of known complications associated with MSA implantation and their respective frequencies. A literature search identified studies reporting on MSA removal, published between January 1, 2008 and August 14, 2024. We queried PubMed, Web of Science, and Embase using all combinations of the terms ‘LINX,’ ‘magnetic sphincter augmentation,’ ‘removal,’ ‘revision,’ and ‘explant.’ Results were imported into Rayyan for evaluation and duplicate removal [5]. We included quantitative studies published in English that reported on MSA explantation and discussed reasons for removal. We excluded animal studies, literature reviews, technical papers, studies unavailable in English, and conference abstracts.

## Results

### Case series

**Fig. 1:**
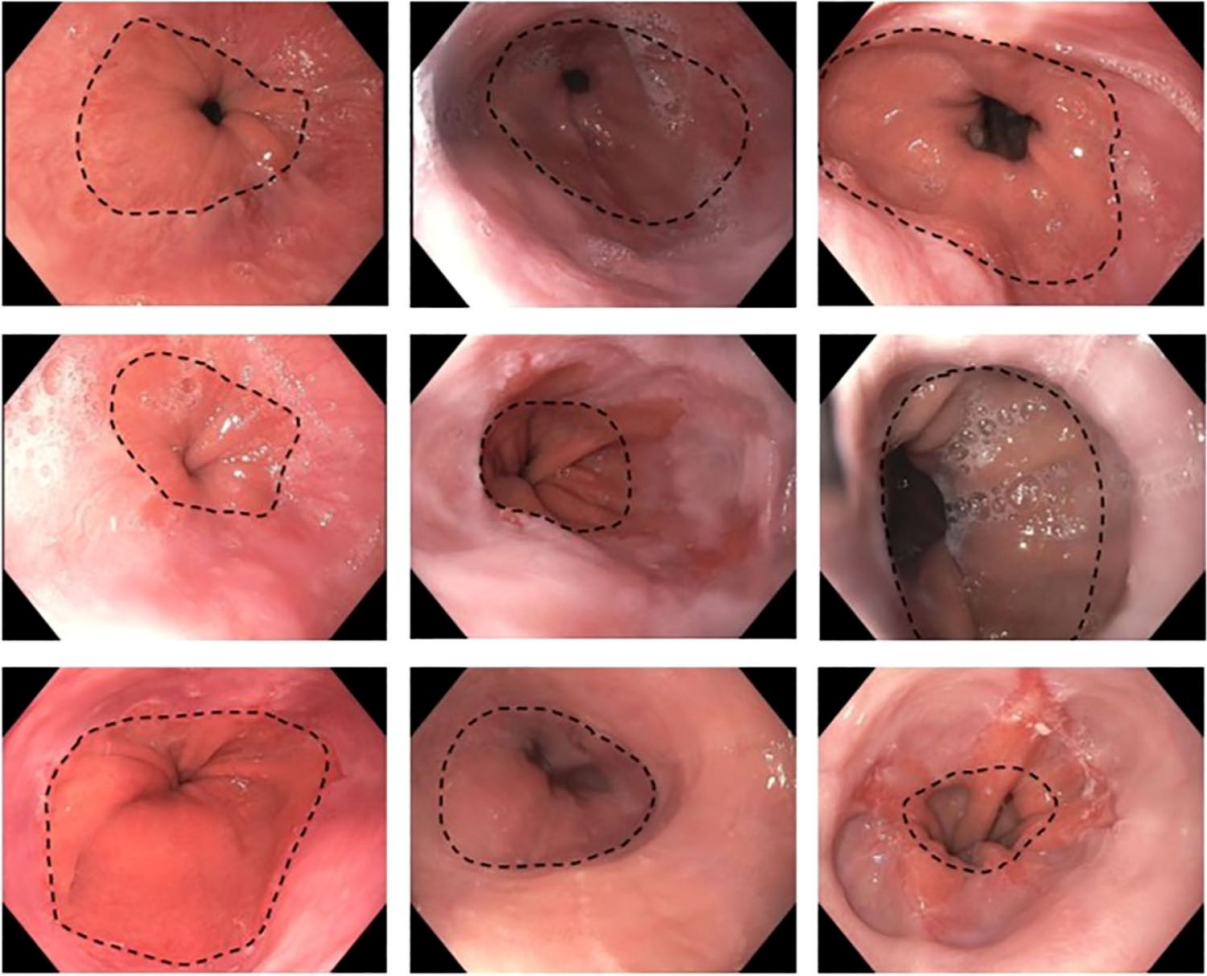
Representative endoscopic images showing slippage of magnetic sphincter augmentation (MSA) device with likely concurrent hiatal hernia. The gastroesophageal junction is identified on maximal insufflation using Z-line and gastric folds as guides. It is marked with black dashed line. This is above the distal narrowing which represents the hiatus and LINX device

A total of 42 patients underwent MSA implantation within our institution. A total of 13 patients who underwent MSA explant were identified (12 were implanted at our institution, and one was implanted at an outside facility). Median age at time of device implant was 47 years (IQR, 39–59). Patients were 92% female (*n* = 12). Median BMI was 31 (IQR 29–33). All patients underwent manometry and objective pre-operative measurement of reflux (Table 1). Ten patients had hiatal hernia and nine of these patients had hiatal hernia repair at time of MSA implantation. Of MSA that were removed, median longevity was 41.1 (IQR, 18.6–58.4) months. Device sizes were as follows: one 12-bead device, five 13-bead devices, two 14-bead devices, four 15-bead devices, and one 16-bead device. A variety of subjective and objective data was gathered in evaluating patients for MSA explant. All patients underwent EGD. A majority of patients underwent at least one endoscopic dilation. The maximum number of endoscopic dilations was four. Three patients underwent 1 dilation; one underwent 2 dilations; four underwent 3 dilations; and one underwent 4 dilations. Four patients did not undergo any endoscopic dilation post-MSA implantation. The most common indication for explant was continued dysphagia after multiple attempts at dilation (Supplemental Table 1). Endoscopic and radiographic data were reviewed for evidence of MSA slippage or hiatal hernia prior to explant. Representative endoscopic images of slipped MSA devices are shown in Fig. 1. Representative radiographs are presented in Supplemental Fig. 1. Operative notes were reviewed to confirm slippage or herniation at time of explant. Ten of 13 patients had hiatal hernia or device slippage. All of these underwent hiatal hernia repair. No cases of erosion were observed. Two cases of device disconnection were observed. One of these occurred after an MRI undertaken for lumbar disk disease (Table 1).

**Table 1:** Clinical characteristics of patients

*Pre-Operative Characteristics*	
Age-years^a^	47 (39–59)
Gender^b^	
Female	12 (92)
Male	1 (8)
BMI^a^	31 (29–33)
Manometric data	
LES length (cm)^a^	2.3 (2.1–3.4)
LES basal pressure (mmHg)^a^	18 (13.7 −31)
LES residual pressure (mmHg)^a^	3.9 (2.7–7.9)
Mean wave amplitude (mmHg)^a^	78 (61–106)
DCI (mmHg cm s)^a^	1684 (854–1852)
Intact swallow (%)^a^	100 (80–100)
DeMeester score	38.4 (29.4–59.8)
*Implant Operation*	
Hiatal hernia repair^b^	9 (69)
Axial displacement of hernia^a^	1.8 (1–2)
Size of implant^b^	
12-bead device	1 (8)
13-bead device	5 (38)
14-bead device	2 (15)
15-bead device	4 (31)
16-bead device	1 (8)
Explant Operation	
Time until explant-months^a^	41.1 (18.6–57.4)
Indication for removal^b^	
Dysphagia	8 (62)
Recurrent reflux	5 (38)
Device disconnection	2 (15)
Hiatal hernia at explant^b^	10 (77)

^a^Median (IQR), ^b^n (%), DCI—distal contractile integral

### Literature review

We focused our review efforts on RCTs, large cohort studies, and case series. The most frequent reasons for removal were dysphagia, followed by reflux [1, 2, 6–46]. Only a minority of studies reported findings of device slippage, hiatal herniation, or migration [1, 2, 6–46]. (Table 2).

**Table 2:** Summary of selected literature

		Herniation				Explant Reasons						
Author	Year	Observed/ Explanted		Implant	Explants	Erosion	Disconnection	Dys-phagia	Reflux	Other ^b^ specified	Other un-specified	Duration of follow up (months) ^c^
Antiporda[6]	2019	0	0	98	5	1	0	1	2	1	0	46 (25–51)
Asti[7]	2017	3	3	164	11	2	0	4	5	0	0	48 (36)
Asti[2]	2023	0	0	130	6	0	1	3	1	1	0	12 (12–24)
Ayazi[8]	2020	24	7	350	18	0	0	18	0	0	0	μ 13.6 SD 10.4
Ayazi[9]	2020	0	0	380	14	0	0	7	0	0	7	μ 11.5 SD 8.7
Ayazi[10]	2020	3	3	553	37	0	2	20	7	8	0	μ 10.3 SD 10.6
Baison[11]	2023	2	2	210	18	2	0	5	9	2	0	22.8 (21.6–31.2) 27.6 (15.6–36) ^d^
Bell[12]	2020	0	0	75	0	0	0	0	0	0	0	12 ^e^
Bellorin[13]	2021	0	0	31	1	0	1	0	0	0	0	Range 1–21
Bologheanu[14]	2022	4	4	357	2	0	0	0	0	2	0	23 (9–36)
Bonavina[15]	2010	0	0	44	1	0	0	1	0	0	0	29.4; Range 7.4–37.6
Bonavina[16]	2013	0	0	100	3	0	0	2	1	0	0	36; Range 12.4–72
Bonavina[17]	2021	0	0	459	11	0	0	5	2	4	0	36 ^e^
Bridges[18]	2022	0	0	106	11	0	0	0	0	0	11	12 ^e^
Broderick[19]	2020	0	0	13	0	0	0	0	0	0	0	μ 20.75 ^e^
Buckley[20]	2018	4	1	200	2	0	0	0	1	1	0	8.6 ^e^
DeMarchi[21]	2021	3	3	27,779	609	27	17	292	125	80	68	Not applicable
Dominguez- Profeta[46]	2021	0	0	68	3	0	0	2	0	1	0	μ 26.6 ^e^
Dunn[22]	2021	5	1	79	5	0	2	2	0	1	0	35.8 (22.8–39.8)
Eriksson[23]	2023	0	0	777	40	0	0	31	9	0	0	Range 5.4–27.2
Eriksson[24]	2023	0	0	131	4	0	0	0	0	0	4	12.4 (1.7)
Ferrari[25]	2021	10	U ^a^	336	32	6	0	6	12	0	8	24 (75); 32 (84) ^d^
Froiio[26]	2023	0	0	397	50	1	1	21	12	9	6	39.5 (53.7)
Ganz[1]	2013	0	0	100	6	0	0	3	1	2	0	36 ^e^
Hawasli[27]	2019	0	0	13	1	0	0	1	0	0	0	μ 26 SD 12
Ibach[28]	2024	0	0	141	6	0	0	3	1	2	0	μ 46.8 ^e^
Khaitan[29]	2023	1	U ^a^	30	2	0	0	2	0	0	0	12 ^e^
Kuckelman[30]	2018	0	0	28	1	0	0	1	0	0	0	μ 14.9 ^e^
Leeds[31]	2020	0	0	99	5	0	0	5	0	0	0	μ 10.2 ^e^
Leeds[32]	2021	0	0	103	9	0	0	0	0	0	9	10; Range 5–15
Louie[33]	2019	0	0	200	5	1	0	2	0	2	0	12 ^e^
Puri[34]	2023	2	U ^a^	202	5	0	1	2	1	1	0	24 (12–36)
Riccardi[35]	2023	0	0	336	19	0	0	19	0	0	0	μ 14.8 SD 7.1
Riva[36]	2019	0	0	67	0	0	0	0	0	0	0	11(8); 13 (3.5) ^d^
Rona[37]	2018	2	1	53	2	0	0	1	1	0	0	μ 19; Range 1–39
Saino [38]	2015	0	0	44	3	0	0	1	1	1	0	60 ^e^
Sarici [39]	2023	0	0	697	42	0	0	0	0	0	42	μ 12.3; SD 3.4
Sarici [40]	2024	0	0	604	32	0	0	24	8	0	0	μ 14.2 SD 7.9
Schwameis [41]	2021	16	7	334	16	1	0	0	0	0	15	μ 13.6 SD 10.4
Schwameis [42]	2018	0	0	68	2	0	0	0	0	2	0	13 (4.2–45)
Schwameis [43]	2018	0	0	68	2	0	0	2	0	0	0	13 (4.2–45)
Tatum [44]	2019	9	9	435	24	2	0	8	13	0	1	μ 28.3; Range 3.9–57.8
Tsai [45]	2020	3	0	118	3	0	0	2	1	0	0	μ 7.8; Range 1–27.7

Summary of key studies reporting magnetic sphincter augmentation (MSA) removal and associated findings. Author (First author, last name); Year; Number of hiatal hernias, migrated or slipped devices reported; Number of these devices removed or revised Number of implants reported; Number of explants reported; Number of explants due to erosion; Number of explants due to device disconnection; Number of explants due to dysphagia; Number of explants due to recurrent reflux; Number of explants due to other specified reasons b. (MRI, pain, regurgitation, unrelated other operation including gastrectomy, esophagectomy, trauma); Number of explants due to other unspecified cause; Duration of follow-up. a Studies where hiatal hernias were reported but it is unknown (U) whether those particular devices were removed. It is assumed they were not removed. C. Reported as median (interquartile range) unless stated otherwise. D. Median and IQR reported separately for 2 groups. μ Mean. SD Standard deviation. E. Measures of data dispersion (i.e., IQR or SD) not provided

Within the context of device slippage, hiatal herniation, or migration, a few studies stand out as particularly relevant. The first is a study of the FDA Manufacturer and User Facility Device Experience (MAUDE) by DeMarchi and colleagues in 2021 [21], the largest study of device complications of all types to date. They reported on 27,779 implants, with 609 subsequent explants, for an explant rate of 2.19%. Reasons for removal were dysphagia (292 devices, 47.9%), GERD (125, 20.5%), abdominal pain (46, 7.9%), erosion (27, 4.4%), device discontinuity (17, 2.8%), vomiting (16, 2.6%), need for MRI (11, 1.8%), gastroparesis (4, 0.7%), device migration (3, 0.5%), and unknown other (68, 11%) [5]. In addition to providing the largest review of device complications, DeMarchi et al. provide a helpful overview of the history of the LINX device including changes in recommendations since the inception of the device. These include changes to the sizing protocol, and the recommendation for complete crural dissection.

In 2020, Ayazi and colleagues published a study which has the most thorough discussion to date on post-MSA hiatal hernias [8]. This study was a retrospective study of 350 patients including 285 MSA implantations in patients who had hiatal hernia. Mean follow-up was 13.6 months, with standard deviation of 10.4 months. They found 24 recurrent hiatal hernias, of which 7 underwent reoperation, in accordance with a classification and management strategy for post-operative hiatal hernias which the authors propose in the study. They recommend endoscopic dilation for small, early hiatal hernia recurrence with device proximal to GEJ, and operative management for larger, symptomatic hiatal hernias and hiatal hernias with device malposition with respect to GEJ. In total from their series of 350 patients, 18 patients underwent explantation [8].

Three other studies explicitly investigated the question of recurrent hiatal hernia after MSA in patients with hiatal hernia [20]. Buckley et al. reported 4 hiatal hernias, found on routine follow-up esophagram out of 200 devices implanted; only one was symptomatic and underwent reoperation. Mean follow-up duration was 8.6 months [20]. Dunn et al. found 5 hiatal hernias out of 79 implants on routine esophagram or EGD of which only 1 was symptomatic and underwent reoperation; median follow-up was 35.8 months (IQR 22.8–39.8) [22]. Rona et al. found 2 hiatal hernias (again by routine esophagram or EGD) out of 53 devices implanted; neither of these patients underwent reoperation with median follow-up of 19 months [37].

Out of the remaining 38 studies considered and presented in Table 2, only 10 studies reported findings of hiatal hernia [7, 10, 11, 14, 25, 29, 34, 41, 44, 45]. As in the previously described studies, hiatal hernias or device slippage were generally noted to occur at low rates.

Across all studies, the explant rate was found to be between 0 and 12.6%, with a median of 4.7% [2, 6–46]. As above, the most frequent reasons for removal were dysphagia, followed by reflux. Other reasons for removal were erosion, device disconnection, other specified reasons (pain, regurgitation, need for MRI, unrelated other operation including gastrectomy, esophagectomy, trauma), and other unspecified reasons [2, 6–46].

## Discussion

The LINX device has significantly advanced GERD surgery, with over 40,000 implants and high patient satisfaction. Since its introduction, optimal use has been refined: sizing now favors a looser fit, early 12-magnet models are no longer produced [47], and recent years show increased use of larger 15- and 16-magnet devices over 13- and 14-magnet devices [21]. These adjustments have reduced the already low rate of device erosion. Hiatal management has also evolved, with full crural dissection now recommended instead of the previously standard minimal dissection [21]. However, based on our experience, there may be opportunities for further improvement.

Our most notable finding is the consistent pattern of MSA device slippage and hiatal hernia recurrence associated with dysphagia or recurrent reflux. This failure pattern is physiologically plausible and aligns with the pattern seen in Nissen fundoplication [6, 48, 49]. Additionally, the higher MSA failure rate that we observe is also comparable to failure rates of other anti-reflux procedures [6, 48, 49]. A recent American Foregut Society (AFS) white paper outlines the components of the anatomic anti-reflux barrier (ARB): (1) the LES and sling fibers, (2) the crural diaphragm, (3) the phrenoesophageal ligament, (4) the gastroesophageal flap valve, (5) intra-abdominal esophageal length, and (6) the acute angle of His [50]. Anti-reflux surgery (ARS) principles aim to restore this structure both functionally and anatomically [51–53]. Hiatal hernia and device slippage, although generally reported as rare, may be underrepresented in studies that do not consistently track hiatal hernia presence. For example, Buckley et al. [20] and Rona et al. [37] defined post-MSA hiatal hernia only with displacements over 2 cm, while Ayazi et al. [8] alone addressed hiatal hernia classification and its impact on post-operative dysphagia and reflux. Even minor device slippage could disrupt the ARB, suggesting that a more thorough investigation into small hiatal hernias in symptomatic patients may reveal more cases.

Another notable feature of our study is duration of follow-up. The median follow-up in this study, 41.1 months, exceeds that of many studies. This likely captures more long-term complications. This likely contributes to the higher removal rate we observed. Longer follow-up may not fully explain the higher removal rate we observed—this is a potential weakness of this study. Other possible reasons for the higher removal rate include under-reporting of device complications. [54–57] Large studies using FDA or manufacturer databases, such as that by DeMarchi et al. [21] and an earlier study of the MAUDE by Smith et al. [58], report only a 2.2% and 2.7% removal rate, respectively, which suggests under-reporting to regulatory agencies and the device manufacturer. Follow-up care can often be handled by non-specialty providers who may not consistently report complications, and patient follow-up can be lost. Another factor may be patient selection; for example, one of our patients had hyperemesis syndrome from marijuana use, likely leading to device slippage and persistent symptoms even post-explant, while another patient’s anxiety likely significantly contributed to their initial and recurrent foregut symptoms. These cases underscore the need for thorough patient education, multidisciplinary management, and careful patient selection to improve outcomes in anti-reflux surgery.

Thus, while effective, the LINX device may face the same hiatal hernia recurrence challenges as other GERD surgeries. Persistent hiatal defects and diaphragm mobility contribute to high recurrence rates [50]. The MSA device's theoretical advantages—enhancing anti-reflux mechanisms without the issues associated with non-relaxing sphincter augmentations like the Nissen or Angelchik—are clear. Its ability to create scar tissue mimics the phrenoesophageal ligament, stabilizing the gastroesophageal junction. However, our findings indicate that the device can still migrate below the gastroesophageal junction, potentially due to its lack of secure fixation. The current placement recommendation, between the esophagus and the posterior vagus nerve, may not provide sufficient stability. The MSA device also does not recreate the acute angle of His or the gastroesophageal flap valve. Alternatives to standard placement warrant further study, such as hiatal repair with esophageal pexy, device fixation to the esophagus, partial cardia or fundus plication, mesh use, or combinations thereof. Although the hiatal anatomy and its functional demands continue to test reconstructive surgery durability, incremental technique improvements, careful patient selection, and thorough lifestyle counseling remain essential for optimal reflux treatment outcomes.

### Limitations

While our study identifies an area for improvement of the LINX system, several limitations should be noted. Firstly, as a single-center study, it may limit the generalizability of our results. Additionally, the retrospective design introduces inherent biases and limitations, such as selection bias and incomplete data capture, which may influence the reliability of our findings. Furthermore, although longer than most, the still limited duration of follow-up for some patients could affect the assessment of long-term outcomes associated with the LINX device. A larger, multi-center prospective study would be beneficial to validate our findings and provide a more comprehensive understanding of the LINX system’s long-term effectiveness and complications.

Another limitation relates to the literature review. Aggregation of data contained in references was not possible as several studies use overlapping patient populations to analyze particular end-points. While this has helped in allowing researchers to make the most out of limited data sets, it means that aggregation would cause double-counting of some patients.

## Conclusion

While the LINX device offers an effective option for GERD management, our findings highlight an important challenge: device slippage and hiatal hernia recurrence. This failure patterns align with other anti-reflux procedures. Refinements in device stabilization techniques may be possible to improve long-term success rates. Additionally, a multidisciplinary approach to patient selection, management, and follow-up may mitigate complications and enhance the overall durability of MSA. Addressing these aspects through continued surgical innovation and rigorous follow-up practices will be essential for advancing GERD treatment outcomes, and in particular with the LINX system.

## Supplementary Information

Below is the link to the electronic supplementary material.

Supplemental Figure 1: Representative radiographs showing slippage of magnetic sphincter augmentation (MSA) device or hiatal hernia

Supplemental Table 1: Indication for explant for each patient
