# Supplementary figures and images for: The failure pattern for the magnetic sphincter augmentation device: a single-institution case series with literature review
Source: Surg Endosc. 2025 Jun 26;39(8):4956–64. doi: 10.1007/s00464-025-11842-x (PMC12287130; doi:10.1007/s00464-025-11842-x)

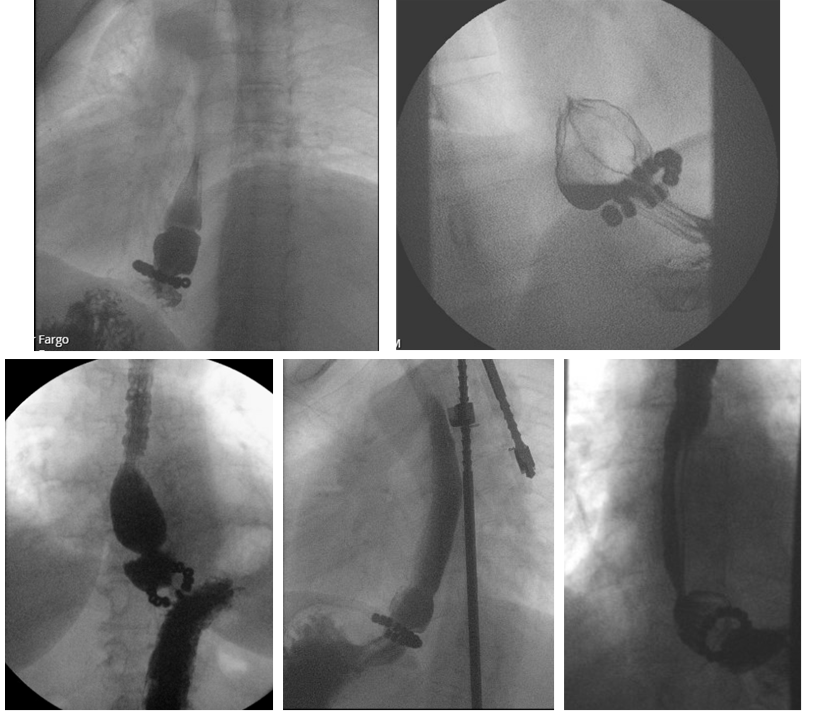

Supplement: Supplementary file 1 — Supplemental Figure 1: Representative radiographs showing slippage of magnetic sphincter augmentation (MSA) device or hiatal hernia [file 464_2025_11842_MOESM1_ESM.png]
