# Supplementary material for: The failure pattern for the magnetic sphincter augmentation device: a single-institution case series with literature review
Source: Surg Endosc. 2025 Jun 26;39(8):4956–64. doi: 10.1007/s00464-025-11842-x (PMC12287130; doi:10.1007/s00464-025-11842-x)
Supplement: Supplementary file 2 — Supplemental Table 1: Indication for explant for each patient [file 464_2025_11842_MOESM2_ESM.docx]

| Supplemental Table 1: Indication for explant for each patient | |
| --- | --- |
| Primary symptom: reflux | |
| Bravo, DeMeester score 19.1 | |
| EGD with LA grade A esophagitis | |
| EGD with LA grade A esophagitis | |
| Mix of reflux and dysphagia | |
| GERDHRQL regurgitation sub-score 8/10, reflux sub-score 16 | |
| GERDHRQL total score 36/75, dissatisfied with disease control | |
| Manometry, impaired clearance, weak LES | |
| Primary symptom: dysphagia | |
| GERDHRQL dysphagia sub-score 7/10 after multiple dilation | |
| Continued subjective complaint of dysphagia after multiple dilation | |
| Esophageal erythema on EGD, subjective complaint of dysphagia | |
| Bravo, DeMeester 38.6 |  |
| Continued subjective complaint of dysphagia after multiple dilation | |
| EGD, LA grade C esophagitis | |
| Continued subjective complaint of dysphagia after multiple dilation | |
